# The role of social cohesion in gingival bleeding levels among adolescents: a cross-sectional study

**DOI:** 10.1590/1807-3107bor-2026.vol40.019

**Published:** 2026-03-30

**Authors:** Raiélli Pivetta MOLETTA, Vitória BARONI, Eduarda da Silveira BORSTMANN, Jessica Klöckner KNORST, Camila Silveira SFREDDO, Thiago Machado ARDENGHI

**Affiliations:** (a) Universidade Federal de Santa Maria - UFSM, School of Dentistry, Department of Stomatology, Santa Maria, RS, Brazil.; (b) Universidade Federal de Pelotas - UFPel, School of Dentistry, Departament of Semiology and Clinic, Pelotas, RS, Brasil.

**Keywords:** Adolescent, Social Capital, Social Cohesion, Observational Study, Gingivitis

## Abstract

This study aimed to evaluate the relationship between social cohesion and gingival bleeding levels in adolescents from Santa Maria, southern Brazil. This cross-sectional study was nested within a cohort study initiated in 2010 with a representative sample of preschool children aged 1 to 5 years in the city. Participants were re-evaluated at ages 11 and 15 years, resulting in a 10-year follow-up period. The current study used data from this follow-up. Social cohesion was assessed at an individual level through questions about perceptions of neighborhood relationships and the frequency of participation in neighborhood gatherings. Gingival bleeding was evaluated clinically using the Community Periodontal Index (CPI). Sociodemographic and clinical variables were considered as potential confounders. Multilevel adjusted Poisson regression was used to assess associations, and results were expressed as rate ratios (RR) with 95% confidence intervals (95%CI). A total of 429 adolescents were included. In the adjusted model, poor perception of neighborhood relationships was associated with higher gingival bleeding levels (RR = 1.08; 95%CI: 1.03–1.13). Adolescents who did not attend local gatherings had even higher bleeding levels (RR = 1.45; 95%CI: 1.34–1.47). Additionally, non-white adolescents, those with lower income, irregular dental visits, and higher plaque levels also showed greater bleeding. Girls had lower gingival bleeding levels compared to boys. Lower social cohesion, reflected by negative neighborhood perceptions and lack of community participation, was associated with higher gingival bleeding in adolescents. Public health strategies aiming to improve adolescent oral health should focus on strengthening neighborhood relationships and promoting active community engagement as key social determinants.

## Introduction

Gingivitis is characterized by an inflammatory response in the gingival tissues due to the accumulation of dental biofilm at the gingival margin, disrupting the balance between bacteria and the host’s immune response.^1^ Gingival bleeding is the most prevalent sign of periodontal disease among Brazilian children and adolescents and an early marker of inflammation.^2^If untreated, gingivitis can progress to periodontitis and tooth loss.^3^ Besides the clinical impacts, gingival bleeding at this age may negatively affect oral health-related quality of life (OHRQoL).^4^

Beyond biological factors, social determinants significantly influence oral health. Social capital, defined as the network of trust and reciprocity within a community, has been associated with better oral health outcomes.^5^ Within the cognitive dimension of social capital is social cohesion, which refers to the strength of relationships and solidarity within communities.^6^ It involves the social bonds that tie individuals together, fostering a sense of belonging, trust, and mutual support.^5-8^ High levels of social cohesion are linked to lower levels of social conflict, greater community well-being, and broader social inclusion, ensuring that all individuals, regardless of background, have access to opportunities.^5,6,9^

While previous research has explored the role of social capital in OHRQoL^10^ and dental caries experience among adolescents,^11^ no studies to date have examined the association between social cohesion and gingival bleeding. Evidence on this topic has largely focused on social capital, and studies in children show that both contextual and individual social capital are associated with lower gingival bleeding levels.^12^ This emphasizes the need to investigate whether similar associations exist when focusing specifically on social cohesion.

Adolescence is a critical transitional phase, marked by major psychosocial changes,^13^ increased autonomy, exposure to broader social environments, and heightened sensitivity to community dynamics, making social cohesion especially important in shaping health behaviors.^14^ Gingival bleeding is the most common sign of periodontal disease in this age group, serving as an early marker of poor oral health and potentially predicting worse periodontal outcomes in adulthood.^15^ Understanding how social cohesion is associated with gingival bleeding during adolescence can provide a better understanding of the social mechanisms that shape oral health and inform preventive strategies targeting behavioral and structural determinants.

Thus, this study aimed to assess the association between social cohesion and gingival bleeding among Brazilian adolescents. We hypothesized that lower levels of individual social cohesion would be associated with higher levels of gingival bleeding.

## Methods

This study adhered to the Strengthening the Reporting of Observational Studies in Epidemiology (STROBE) guidelines.

### Study design and population

This cross-sectional study was nested within a 10-year cohort study conducted in Santa Maria, southern Brazil. The original cohort was established in 2010 during the National Child Vaccination Day. At the time, the city had an estimated population of 263,403, which included 27,520 children aged up to 5 years.^16^ Children aged 1 to 5 years were systematically selected from 15 health centers equipped with dental chairs, covering the city’s eight administrative regions, resulting in a sample of 639 children. Participants were subsequently re-evaluated in 2012, 2017, and 2020. The present analysis used data from the last follow-up (Figure). Detailed information on the cohort methodology has been previously published.^10^

**Figure:**
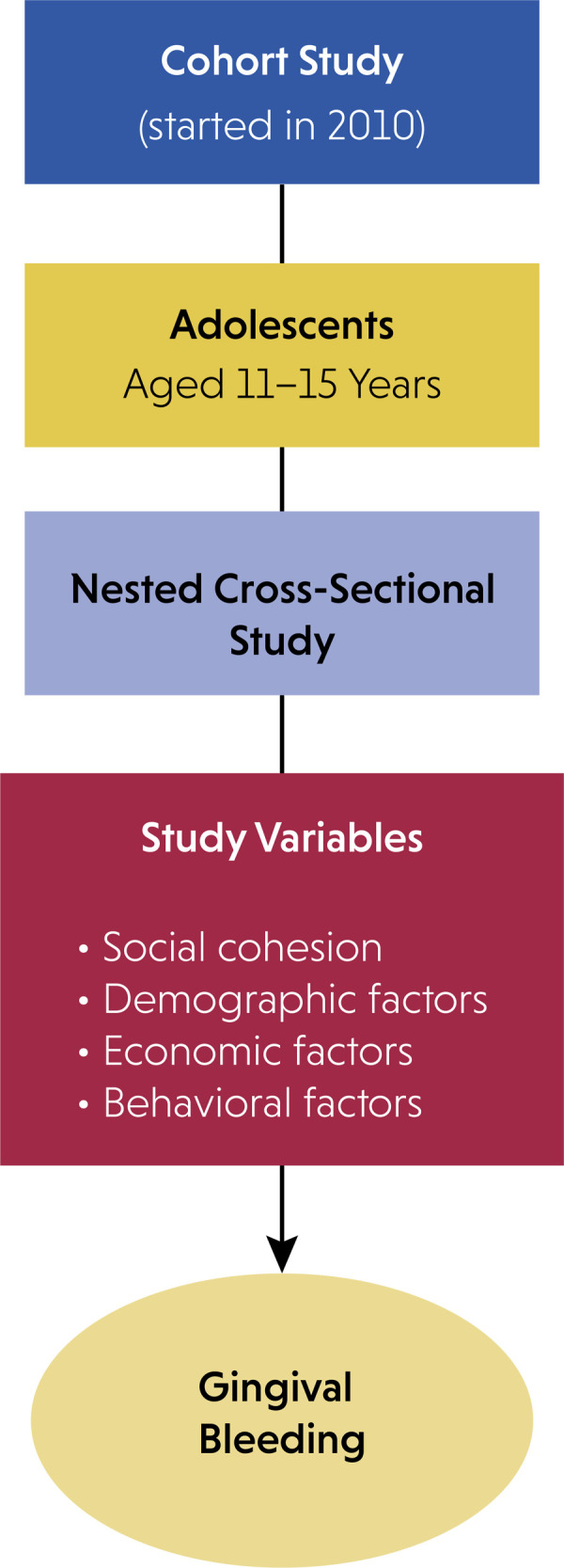
Schematic diagram of the study design.

To minimize nonresponse, strategies included updating contact information by telephone, locating adolescents through school enrolment lists, and using social media when necessary. A post hoc power analysis confirmed the adequacy of the sample size, considering a significance level of 5% and a 95% confidence interval. Based on the mean difference in gingival bleeding between adolescents with low versus high social cohesion, the statistical power was determined to be 100%.

### Data collection and variables

The adolescents were evaluated in their schools or residences after authorization from the caregivers. Clinical evaluations were conducted individually under natural light using gauze, a periodontal probe (Community Periodontal Index [CPI]; “ballpoint”), and a dental mirror. Gingival bleeding was assessed at six sites per tooth^17^ based on the CPI, and rated as either healthy (0) or exhibiting bleeding (1). Additionally, visible plaque was assessed for each tooth and recorded as either present or absent. For analytical purposes, the mean percentage of dental sites presenting gingival bleeding was considered. Before data collection, all examiners underwent a standardized training process to ensure consistency and reliability in clinical assessments. The training included a theoretical session conducted by a leading researcher in the field, followed by clinical standardization for periodontal evaluation. A total of 5 examiners were trained for this study.

Social cohesion was assessed using proxy questions derived from the cognitive dimension of the Short Adapted Social Capital Assessment Tool (S-ASCAT), which encompasses trust and social cohesion. They are part of a validated instrument developed by Harpham et al.^18^ and later adapted for adolescents by Story et al.^19^ In our study, only social cohesion items were applied, which encompass aspects related to neighborhood relationships and participation in community meetings, as follows: a) “In the past 12 months, have you joined other people in your neighborhood or community to address important issues of general interest?” with response options: 0 = yes or 1 = no; and b) “Do most people in your neighborhood generally have good relationships with each other?” with response options: 0 = yes, 1 = sometimes, or 2 = no.^18,19^

Demographic and socioeconomic variables included sex (male or female), age (recorded in years and dichotomized based on the sample mean), and self-reported skin color, classified as white or non-white according to the Brazilian Institute of Geography and Statistics.^16^ Skin color was determined using the question “What race do you consider yourself?” and subsequently dichotomized into “white” and “non-white” (including black, brown, yellow, or indigenous individuals). The socioeconomic variable considered was family income, recorded in Brazilian reais (R$) and categorized based on the Brazilian minimum wage (BMW) as either < 1 BMW or ≥ 1 BMW. At the time of data collection, one BMW was equivalent to USD$ 250. Use of dental services was assessed through the question: “In the last year, how many times have you been to the dentist?” with response options: none, once, twice, three times or more, or never been. For analytical purposes, responses were dichotomized into “regular visits” (once, twice, or three times or more) and “no regular visits” (none or never been).^17^

### Ethical aspects

This study was approved by the Committee for Ethics in Research of the Federal University of Santa Maria (protocol number CAAE 54257216.1.0000.5346). In accordance with ethical standards for research involving minors, parents or legal guardians signed an informed consent form, and adolescents provided written assent to participate in all phases of the study. Furthermore, the study was conducted according to National Health Council of Brazil.

### Data analysis

Data analysis was performed using the statistical software STATA 17.0 (Stata Corporation, College Station, USA). Descriptive analysis was conducted to assess the characteristics of the sample. The primary outcome of the study was the mean percentage of dental sites exhibiting gingival bleeding. A descriptive analysis was also performed to evaluate sample characteristics based on mean gingival bleeding. Unadjusted and adjusted Poisson regression models were used to examine the association between various variables and gingival bleeding. Variables with a p-value < 0.20 in the unadjusted analysis were included in the adjusted models. Results are presented as rate ratio (RR) with 95% confidence interval (95%CI). Model fit was assessed using Pearson’s chi-square statistic, deviance, and the Akaike Information Criterion (AIC), allowing comparisons between crude and adjusted models.

## Results

A total of 429 participants were included in this study, representing 67.1% of the original cohort. The remaining individuals were excluded due to refusal to participate, relocation to other cities, or inability to be located.

Demographic, socioeconomic, and oral health-related characteristics of the sample are presented in Table 1. The sample was balanced in terms of sex, with similar proportions of female and male adolescents. The mean age of the participants was 12.6 years (SD = 1.36), and 51.5% identified as non-white. Regarding socioeconomic characteristic, the mean household income was R$2,388.21 (SD = 2,225.44). Additionally, 79.1% of the adolescents reported regular dental visits. Regarding social cohesion, 57.8% of adolescents perceived their neighborhood relationships as positive, while 42.2% considered them poor. Only 16.8% reported attending neighborhood meetings. Clinical characteristics revealed a mean of 0.92 (SE 0.1) and 0.11 (SE 0.1) sites with visible plaque and gingival bleeding, respectively.

**Table 1:** Demographic, socioeconomic, and oral health-related variables, Santa Maria, Brazil (n = 429).

Variables	n (%)
Demographic and socioeconomic characteristics	
Sex	
Male	209 (49.8)
Female	220 (50.2)
Age (years)	
< 12	189 (43.5)
> 12	240 (56.1)
Skin color	
White	215 (48.5)
Non-white	211 (51.5)
Household income (BMW)	
> 1	264 (70.8)
< 1	110 (29.2)
Regular visits to the dentist	
Yes	352 (79.1)
No	77 (20.9)
Social cohesion	
Perception of neighborhood relationships	
Good	240 (57.8)
Poor	189 (42.2)
Attends neighborhood meetings	
Yes	61 (16.8)
No	367 (83.2)
Clinical characteristics	
Visible plaque (mean [SE])	0.92 (0.1)
Outcome	
Gingival bleeding (mean [SE])	0.11 (0.1)

SE: standard error; BMW: Brazilian minimum wage.

Table 2 presents the characteristics of the sample according to mean gingival bleeding. Regarding social cohesion, a poor perception of neighborhood relationships was associated with a higher mean of sites with gingival bleeding (22.1; SE 3.1). Similarly, adolescents who did not attend neighborhood meetings exhibited a higher mean of sites with gingival bleeding (mean 22.3; SE 2.6).

**Table 2:** Sample characteristics according to mean of sites with gingival bleeding, Santa Maria, Brazil (n = 429).

Variables	Gingival bleeding
	Mean (SE)
Demographic and socioeconomic characteristics	
Sex	
Male	28.0 (4.1)
Female	14.1 (1.5)
Age (years)	
< 12	17.3 (2.8)
> 12	23.8 (17.1)
Skin color	
White	16.8 (2.2)
Non-white	25.2 (3.9)
Household income (BMW)	
> 1	18.6 (2.2)
< 1	24.2 (4.8)
Regular visits to the dentist	
Yes	18.8 (1.8)
No	29.5 (8.2)
Social cohesion	
Perception of neighborhood relationships	
Good	20.2 (3.2)
Poor	22.1 (3.1)
Attends neighborhood meetings	
Yes	14.7 (3.1)
No	22.3 (2.6)
Clinical characteristics	
Visible plaque (mean [SE])	0.20*

SE: standard error; BMW: Brazilian minimum wage; *p-value < 0.01 (Pearson correlation).

Unadjusted and adjusted Poisson regression analyses are presented in Table 3. In the unadjusted analysis, adolescents with a negative perception of neighborhood relationships and those who did not attend neighborhood meetings showed higher levels of gingival bleeding (p < 0.05). Additionally, sex, age, skin color, household income, use of dental services, and dental plaque were associated with gingival bleeding (p < 0.05). After adjustment, adolescents with a negative perception of neighborhood relationships had about 0.8% higher mean of sites with gingival bleeding (RR = 1.08; 95%CI: 1.03–1.13). Lack of participation in neighborhood meetings was also associated with a higher mean of sites with gingival bleeding (RR = 1.45; 95%CI: 1.34–1.47), being about 45% higher in this group. Older age, non-white skin color, low household income, and the presence of visible plaque were also associated with higher levels of gingival bleeding. In contrast, female adolescents had lower levels of gingival bleeding.

**Table 3:** Unadjusted and adjusted analysis of the association between variables and gingival bleeding, Santa Mari, Brazil (n = 429).

Variables	Unadjusted	p-value	Adjusted
	RR (95% CI)		RR (95% CI)
Demographic and socioeconomic characteristics			
Sex		< 0.01	1 (reference)
Male	1 (reference)		0.80 (0.76–0.84)*
Female	0.69 (0.66–0.72)		
Age (years)	(reference)	< 0.01	
< 12	1.14 (1.12–1.16)		1 (reference)
> 12			1.35 (1.26–1.39)*
Skin color		< 0.01	
White	1 (reference)		1 (reference)
Non-white	1.47 (1.40–1.154)		1.35 (1.29–1.42)*
Household income (BMW)		< 0.01	1 (reference)
> 1	1 (reference)		1.10 (1.04–1.17)*
< 1	1.15 (1.08–1.22)		
Regular visits to the dentist		< 0.01	1 (reference)
Yes	1 (reference)		1.04 (0.98–1.11)
No	1.17 (1.11–1.124)		
Social cohesion			
Perception of neighborhood relationships		< 0.01	
Good	1 (reference)		1(reference)
Poor	1.07 (1.02–1.12)		1.08 (1.03–1.13)*
Attends neighborhood meetings		< 0.01	
Yes	1 (reference)		1 (reference)
No	1.55 (1.44–1.67)		1.45 (1.34–1.47)*
Clinical characteristics			
Visible plaque	10.29 (8.18–12.95)	< 0.01	8.75 (6.96-11.02)*
Goodness of fit	Crude		Adjusted
Pearson χ^2^	14390.51		6696.34
Deviance	13.099.175		7.799.255
AIC	3.874.514		77.812.546

BMW: Brazilian minimum wage; RR: rate ratio; CI: confidence interval; AIC: Akaike Information Criterion; *p-value < 0.05.

## Discussion

This study aimed to evaluate the association between social cohesion and gingival bleeding among adolescents. Our findings support the hypothesis that adolescents with lower levels of social cohesion, such as poorer perceptions of neighborhood relationships and lack of participation in neighborhood meetings, exhibited higher mean of gingival bleeding. To the best of our knowledge, this is the first study to assess this association in this stage of life. In addition to social cohesion, sociodemographic factors such as sex, age, skin color, and household income were associated with gingival bleeding. Nevertheless, after adjusting for these variables, the association between social cohesion and gingival bleeding remained significant, indicating that social cohesion exerts an independent effect.

Several approaches attempt to explain how social capital can influence oral health conditions. Behavioral theory emphasizes that high levels of social capital facilitate the dissemination of knowledge and healthy habits through informal social norms and peer pressure. Psychosocial theory suggests that more cohesive societies with greater social capital provide enhanced social support, a sense of security, and belonging among individuals, acting as a protective factor against psychosocial stress and its negative effects on health.^7,8^ The theory of access to healthcare services and public health policies posits that communities with high levels of social capital are more actively engaged in advocating for access to high-quality social and healthcare services.^7,8^ Thus, communities with higher levels of social cohesion among residents are likely to present better health habits, greater social support, increased access to healthcare services,^20^ and, consequently, better oral health outcomes^7,10,20^, such as lower gingival bleeding indices.

Our results demonstrated that individuals with a poorer perception of neighborhood relationships exhibited higher mean of gingival bleeding. The perception of interpersonal relationships among individuals living in the same neighborhood may constitute the cognitive dimension of social cohesion, reflecting aspects of interpersonal trust and a sense of belonging to the place where individuals live^5,7,8^. Such trust and belonging may influence adolescents’ health behaviors, including their approach to hygiene and seeking dental care. Adolescents who feel more connected and supported by their community might be more likely to engage in preventive dental care and maintain better oral hygiene practices,^20^ thereby reducing the likelihood of gingival bleeding. In contrast, those with poorer perceptions of neighborhood relationships may lack this support, and, consequently, have higher levels of gingival bleeding. Moreover, greater social cohesion is associated with a sense of belonging and reduced psychosocial stress. Lower levels of stress may, in turn, reduce inflammatory responses that contribute to gingival bleeding.^8,12^ These mechanisms provide a plausible link between distal social determinants and the outcomes assessed in our study.

Additionally, our findings showed that adolescents who participated more frequently in neighborhood meetings had a lower mean of gingival bleeding. The participation in neighborhood meetings may constitute the structural dimension of social cohesion, or its quantitative component, which refers to the extent and intensity of individuals’ participation in activities and other forms of social engagement, i.e., the quantity and structure of social networks that individuals have.^5,7,8^ Adolescents who are more engaged in these community activities may benefit from stronger social support networks, which can positively influence health behaviors such as adherence to regular dental visits or improved oral hygiene practices.^20^ This engagement might contribute to lower mean levels of gingival bleeding, as socially engaged individuals may have better access to resources and health information.

It is important to highlight that the effect of the frequency of participation in neighborhood meetings on gingival bleeding was greater than the effect of the perception of neighborhood relationships. These findings contradict previous studies that demonstrated the cognitive dimension of social cohesion plays a more significant role in oral health conditions, such as OHRQoL during adolescence.^10^ Our findings may be explained by the fact that participation in neighborhood meetings facilitates contact and the formation of social bonds among individuals living in the same neighborhood. Furthermore, we hypothesize that this participation in social activities, in addition to increasing the frequency of social networks, also fosters the development of trust and social cohesion, creating a more positive social environment. This, in turn, impacts better oral health outcomes through various pathways in this population, including access to dental services.^5,7,8,20^

Our results also showed that females had a lower mean of gingival bleeding compared to males, which is consistent with a previous study that explains how males tend to exhibit poorer hygiene habits and oral health behaviors.^21^ Older adolescents also exhibited a higher mean of gingival bleeding, in accordance with previous studies.^22-24^ During adolescence, the significant increase in steroid hormone levels affects the inflammatory status of the gingiva without a concomitant increase in plaque levels.^1,25,26^ Non-white adolescents also had higher mean of gingival bleeding compared to white adolescents, following previous studies that highlight racial inequities in oral health conditions.^27,28^ Similarly to previous studies, lower household income was also associated with a higher mean of gingival bleeding.^29-31^ Income is a recognized indicator of socioeconomic status,^29,32^ which can affect oral health due to lack of material resources, limited access to healthcare services, unhealthy behaviors, and increased psychosocial stress.^29,33^ Finally, the presence of visible plaque was strongly associated with the outcome. Gingival bleeding is the most common sign of periodontal diseases in adolescence,^2^ primarily caused by the accumulation of dental biofilm.^1,34^ Thus, the plaque‐induced gingival inflammatory conditions require the presence of dental plaque coupled with clinical signs and symptoms of gingival inflammation in periodontal tissues.

This study has some limitations. The major limitation is the use of single questions to measure social cohesion, which may not fully capture this complex construct. However, the variables were assessed through indicators such as the perception of neighborhood relationships and the frequency of participation in neighborhood meetings, which have been used in previous studies.^10,35^ Additionally, the periodontal examination was conducted under field conditions, which may have influenced the assessment of gingival bleeding. However, the study followed the World Health Organization (WHO) recommendations for epidemiological studies, and the CPI is a valid and widely used method.^19^ Although this was a cross-sectional study nested within a longitudinal framework, we cannot establish causal relationships between social cohesion and gingival bleeding. Therefore, future longitudinal research is needed to better understand the effects of social cohesion on gingival bleeding levels. Additionally, self-reported measures are susceptible to social desirability and recall bias, which could have led to overestimation of social cohesion or misreporting of dental visits.

This study also has strengths. Our research addresses a gap in the literature by exploring the role of social cohesion in gingival bleeding levels during adolescence, a period marked by significant behavioral and psychosocial changes. Understanding the challenges faced by this age group is relevant for developing health strategies that can improve long-term health outcomes. In practice, strengthening social cohesion among adolescents may contribute to better oral health outcomes by fostering supportive environments, increasing access to health information, and promoting healthier behaviors.

In the context of the city investigated, strengthening social cohesion could be fostered through initiatives that promote trust, mutual support, and active participation among residents. Examples include organizing cultural, sports, and educational events in public spaces; improving the safety, accessibility, and quality of shared community areas; and encouraging civic engagement in neighborhood decision-making processes. These locally driven actions have the potential to enhance interpersonal connections, foster a stronger sense of belonging, and indirectly promote healthier behaviors, including those related to oral health.

From a public health perspective, strengthening social cohesion may contribute to improved oral health outcomes in youth. School- and community-based programs that promote peer support, extracurricular activities, and civic engagement could foster supportive environments conducive to healthy behaviors. In Brazil, such strategies could be integrated into existing initiatives, such as the School Health Program,^36^ the Family Health Strategy,^37^ and the Social Assistance Reference Centers (CRAS),^38^ thereby enhancing their capacity to reduce inequalities and promote oral health. Aligning oral health promotion with efforts to build stronger community bonds may not only reduce gingival bleeding prevalence but also improve overall well-being and equity in adolescent populations.

## Conclusion

Lower social cohesion was associated with higher levels of gingival bleeding among adolescents. These findings highlight the importance of incorporating distal social determinants, such as social cohesion, in understanding and addressing oral health outcomes. Public health policies and preventive programs should therefore incorporate strategies that foster community engagement, strengthen neighborhood ties, and integrate oral health promotion within broader social and health initiatives. Such comprehensive approaches are essential to reduce oral health disparities and enhance adolescents’ overall quality of life.

## Data Availability

The datasets generated during and/or analyzed during the current study are available from the corresponding author on reasonable request.

## References

[1] Murakami S, Mealey BL, Mariotti A, Chapple IL (2018). Dental plaque-induced gingival conditions. J Periodontol.

[2] Ministério da Saúde (BR) (2023). SB Brasil 2023: Pesquisa Nacional de Saúde Bucal: resultados
principais.

[3] Wang Y, Zhuo L, Yang S, Dong C, Shu P (2025). Burden of periodontal diseases in young adults. Sci Rep.

[4] Tomazoni F, Zanatta FB, Tuchtenhagen S, Rosa GN, Del Fabro JP, Ardenghi TM (2014). Association of gingivitis with child oral health-related quality
of life. J Periodontol.

[5] Moore S, Kawachi I (2017). Twenty years of social capital and health research: a
glossary. J Epidemiol Community Health.

[6] Fonseca X, Lukosch S, Brazier F (2019). Social cohesion revisited: a new definition and how to
characterize it. Innovation (Abingdon).

[7] Knorst JK, Vettore MV, Ardenghi TM (2022). Social capital and oral health promotion: Past, present, and
future challenges. Front Oral Health.

[8] Rouxel PL, Heilmann A, Aida J, Tsakos G, Watt RG (2015). Social capital: theory, evidence, and implications for oral
health. Community Dent Oral Epidemiol.

[9] Rostila M (2010). The facets of social capital. J Theory Soc Behav.

[10] Knorst JK, Vettore MV, Brondani B, Emmanuelli B, Ardenghi TM (2023). The different roles of structural and cognitive social capital on
oral health-related quality of life among adolescents. Int J Environ Res Public Health.

[11] Knorst JK, Tomazoni F, Sfreddo CS, Vettore MV, Hesse D, Ardenghi TM (2022). Social capital and oral health in children and adolescents: A
systematic review and meta-analysis. Community Dent Oral Epidemiol.

[12] Ferreira DM, Knorst JK, Menegazzo GR, Bolsson GB, Ardenghi TM (2021). Effect of individual and neighborhood social capital on gingival
bleeding in children: A 7-year cohort study. J Periodontol.

[13] World Health Organization (2005). Nutrition in adolescence: issues and challenges for the health
sector.

[14] Viner RM, Ozer EM, Denny S, Marmot M, Resnick M, Fatusi A (2012). Adolescence and the social determinants of health. Lancet.

[15] Hashim NT, Babiker R, Padmanabhan V, Ahmed AT, Chaitanya NC, Mohammed R (2025). The global burden of periodontal disease: a narrative review on
unveiling socioeconomic and health challenges. Int J Environ Res Public Health.

[16] Instituto Brasileiro de Geografia e Estatística (2010). Título do documento.

[17] World Health Organization (2013). Oral health surveys: basic methods.

[18] Harpham T, Grant E, Thomas E (2002). Measuring social capital within health surveys: key
issues. Health Policy Plan.

[19] Story WT, Taleb F, Ahasan SM, Ali NA (2015). Validating the measurement of social capital in Bangladesh: a
cognitive approach. Qual Health Res.

[20] Knorst JK, Brondani B, Vettore MV, Hesse D, Mendes FM, Ardenghi TM (2022). Pathways between social capital and oral health from childhood to
adolescence. J Dent Res.

[21] Östberg AL, Halling A, Lindblad U (1999). Gender differences in knowledge, attitude, behavior and perceived
oral health among adolescents. Acta Odontol Scand.

[22] Lawal FB, Dosumu EB (2021). Self-reported and clinically evident gingival bleeding and impact
on oral health-related quality of life in young adolescents: a comparative
study. Malawi Med J.

[23] Silva PL, Barbosa TS, Amato JN, Montes AB, Gavião MB (2015). Gingivitis, psychological factors and quality of life in
children. Oral Health Prev Dent.

[24] Lu HX, Wong MC, Lo EC, McGrath C (2011). Trends in oral health from childhood to early adulthood: a life
course approach. Community Dent Oral Epidemiol.

[25] Chen H, Zhang R, Cheng R, Xu T, Zhang T, Hong X (2020). Gingival bleeding and calculus among 12-year-old Chinese
adolescents: a multilevel analysis. BMC Oral Health.

[26] Mariotti A, Mawhinney M (2013). Endocrinology of sex steroid hormones and cell dynamics in the
periodontium. Periodontol 2000.

[27] Knack KC, Sabadin CE, Boclin KL, Oltramari ES, Portilio MN, Rigo L (2019). Periodontal conditions in adolescents and young Brazilians and
associated factors: cross-sectional study with data from the Brazilian oral
health survey, 2010. J Indian Soc Periodontol.

[28] Bastos JL, Celeste RK, Paradies YC (2018). Racial inequalities in oral health. J Dent Res.

[29] Sfreddo CS, Moreira CH, Celeste RK, Nicolau B, Ardenghi TM (2019). Pathways of socioeconomic inequalities in gingival bleeding among
adolescents. Community Dent Oral Epidemiol.

[30] Koga R, Herkrath AP, Vettore MV, Herkrath FJ, Rebelo Vieira JM, Pereira JV (2020). The role of socioeconomic status and psychosocial factors on
gingivitis in socially disadvantaged adolescents. J Periodontol.

[31] Tomazoni F, Vettore MV, Zanatta FB, Tuchtenhagen S, Moreira CH, Ardenghi TM (2017). The associations of socioeconomic status and social capital with
gingival bleeding among schoolchildren. J Public Health Dent.

[32] Solar O, Irwin A (2010). A conceptual framework for action on the social determinants of
health.

[33] Moor I, Spallek J, Richter M (2017). Explaining socioeconomic inequalities in self-rated health: a
systematic review of the relative contribution of material, psychosocial and
behavioural factors. J Epidemiol Community Health.

[34] Chapple IL, Mealey BL, Van Dyke TE, Bartold PM, Dommisch H, Eickholz P (2018). Periodontal health and gingival diseases and conditions on an
intact and a reduced periodontium: Consensus report of workgroup 1 of the
2017 World Workshop on the Classification of Periodontal and Peri-Implant
Diseases and Conditions. J Clin Periodontol.

[35] Knorst JK, Vettore MV, Brondani B, Emmanuelli B, Paiva SM, Ardenghi TM (2022). Impact of community and individual social capital during early
childhood on oral health-related quality of life: a 10-year prospective
cohort study. J Dent.

[36] Ministério da Saúde (BR) (2022). Programa Saúde na Escola: caderno do gestor do PSE.

[37] Pucca GA, Gabriel M, Araujo ME, Almeida FC (2015). Ten years of a National Oral Health Policy in Brazil: innovation,
boldness, and numerous challenges. J Dent Res.

[38] Ministério da Saúde (BR), Centro de Referência da Assistência Social (CRAS) (2022). Brasília, df: Rede de Assistência e Proteção Social.

